# Cardio-Ankle Vascular Index as an Arterial Stiffness Marker Improves the Prediction of Cardiovascular Events in Patients without Cardiovascular Diseases

**DOI:** 10.3390/jcdd9110368

**Published:** 2022-10-25

**Authors:** Yuko Okamoto, Toru Miyoshi, Keishi Ichikawa, Yoichi Takaya, Kazufumi Nakamura, Hiroshi Ito

**Affiliations:** 1 Department of Cardiovascular Medicine, Okayama University Graduate School of Medicine, Dentistry and Pharmaceutical Sciences, Okayama 700-8558, Japan; 2 Department of Medical Technology, Kawasaki University of Medical Welfare, Kurashiki 701-0193, Japan

**Keywords:** arterial stiffness, cardio-ankle vascular index, cardiovascular events, risk factors

## Abstract

Several studies have reported that the cardio-ankle vascular index (CAVI), a non-invasive measurement of arterial stiffness, is associated with the incidence of cardiovascular events. We investigated whether adding CAVI to a risk score improves the prediction of cardiovascular events in the setting of primary prevention. This retrospective observational study included consecutive 554 outpatients with cardiovascular disease risk factors but without known cardiovascular disease (68 ± 9 years, 64% men). The CAVI was measured using the VaSera vascular screening system. Major adverse cardiovascular events (MACE) included cardiovascular death, myocardial infarction, stroke, hospitalization for heart failure, and coronary revascularization. During a median follow-up of 4.3 years, cardiovascular events occurred in 65 patients (11.7%). Multivariate Cox analysis showed that abnormal CAVI (>9.0) was significantly associated with the incidence of MACE (hazard ratio 2.31, 95% confidence interval 1.27–4.18). The addition of CAVI to the Suita score, a conventional risk score for coronary heart disease in Japan, significantly improved the C statics from 0.642 to 0.713 (*p* = 0.04). In addition to a conventional risk score, CAVI improved the prediction of cardiovascular events in patients with cardiovascular disease risk factors but without known cardiovascular diseases.

## 1. Introduction

Arterial stiffness is closely associated with the risk of cardiovascular disease (CVD) and mortality [1]. Carotid-femoral pulse wave velocity (PWV) has been reported to be associated with an increased risk of first cardiovascular events in the general population and improved risk prediction when added to standard risk factors [2]. Brachial-ankle PWV can be performed more easily than carotid-femoral PWV measurement, while both carotid-femoral PWV and brachial-ankle PWV are affected by blood pressure [1,3], which is an important confounding factor for CVD. To overcome this limitation, the cardio-ankle vascular index (CAVI), a marker of arterial stiffness based on stiffness parameter β, was developed in Japan in 2004 [4]. CAVI can be obtained automatically by wrapping pressure cuffs around the upper and lower legs and is less dependent on blood pressure. Previous studies have reported the association between a greater CAVI and a high incidence of cardiovascular events in patients with diabetes mellitus, obesity, and several CVD risk factors [5,6,7,8,9,10,11,12,13,14,15,16].

The measures of arterial stiffness benefit the prevention of cardiovascular disease, although they have not been widely incorporated into routine clinical practice. To facilitate the use of the CAVI, we proposed a criterion for CAVI based on the expert consensus from the Physiological Diagnosis Criteria for Vascular Failure Committee of the Japan Society for Vascular Failure [17]. We set three ranges in the document: normal range (CAVI ≤ 8.0), borderline range (8.0 < CAVI ≤ 9.0), and abnormal range (CAVI > 9.0). Abnormal CAVI was considered a cutoff value for discriminating the presence of cardiovascular disease or the risk of future cardiovascular events. However, the cutoff value for CAVI has not been adequately validated.

For the risk assessment of CVD, pooled cohort risk equations were introduced by the American College of Cardiology [18] and the European Society of Cardiology [19] to estimate the 10-year atherosclerotic cardiovascular disease. The Suita score was proposed and validated to estimate the 10-year risk of coronary heart disease in the Japanese population [20]. These risk scores are beneficial for assessing patient risk stratification in the setting of primary prevention. As CAVI has been reimbursed by insurance in Japan, its measurement has been included in routine clinical practice. However, the usefulness of CAVI, in addition to the Suita score, has not yet been evaluated. 

This study aimed to investigate [1] whether abnormal CAVI (>9.0) is a good predictor of cardiovascular events in patients with CVD risk factors but without known CVD and [2] whether CAVI offers incremental value in addition to the Suita score for predicting cardiovascular events in a retrospective cohort.

## 2. Methods

### 2.1. Study Population

This retrospective, single-center cohort study evaluated the impact of CAVI on prognosis. We enrolled 554 outpatients between May 2012 and December 2016. They had no history of CVD but had at least one CVD risk factor and were referred to our hospital for examination of coronary artery disease. Patients were excluded for the following reasons: peripheral artery disease, defined as ankle–brachial pressure index < 0.9, left ventricular ejection fraction < 50%, a history of CVD, atrial fibrillation, and hemodialysis. Hypertension was defined as systolic blood pressure ≥ 140 mmHg, diastolic blood pressure ≥ 90 mmHg, and/or the use of antihypertensive medication. Diabetes mellitus was defined as a fasting blood glucose concentration of 126 mg/dL and/or the use of insulin or oral hypoglycemic medication. Dyslipidemia was defined as low-density lipoprotein cholesterol (LDL-C) ≥ 140 mg/dL, triglyceride ≥ 150 mg/dL, high-density lipoprotein cholesterol (HDL-C) < 40 mg/dL, and/or the use of antidyslipidemic medication. The estimated glomerular filtration rate (eGFR) was calculated using the Modification of Diet in Renal Disease equation with the Japanese coefficient [21].

This study was conducted in accordance with the principles of the Declaration of Helsinki and approved by the ethics committees of the Okayama University Graduate School of Medicine, Dentistry, and Pharmaceutical Sciences. The requirement for informed patient consent was waived owing to the low-risk nature of the study and the inability to obtain consent directly from all study subjects.

### 2.2. Measurement of CAVI

Arterial stiffness was evaluated using CAVI, as previously described [6]. After a 5-min rest and with the subject seated, extremity blood pressure was measured using the oscillometric method. CAVI was measured automatically using a VaSera vascular screening system (Fukuda Denshi, Tokyo, Japan) from the measurement of blood pressure and pulse wave velocity (PWV) while monitoring the electrocardiogram and heart sounds. PWV was calculated by dividing the distance from the aortic valve to the ankle artery by the sum of the time between the aortic valve closing sound and the notch of the brachial pulse wave and the time between the rise of the brachial pulse wave and the rise of the ankle pulse wave. CAVI was determined using the following equation: CAVI = a[(2ρ/ΔP) × In(Ps/Pd) × PWV^2^] + b, where Ps and Pd are the systolic and diastolic blood pressures, respectively, PWV is the pulse wave velocity between the heart and ankle, ΔP is Ps − Pd, ρ is blood density, and a and b are constants. The averages of the right and left CAVI were used for the analysis. Patients were classified into three groups based on CAVI levels, as previously described [17]: normal group (CAVI ≤ 8.0), borderline group (8.0 < CAVI ≤ 9.0), and abnormal group (CAVI > 9.0).

### 2.3. The Suita Score

The Suita score has been used to predict the risk of CVD development; hence we used the Suita score in this study [20]. The Suita score is an established CVD risk score based on risk factor categories for predicting coronary heart disease in the Japanese population. According to a previous report, the Suita score LDL-C version was calculated using age, sex, HDL-C and LDL-C levels, systolic blood pressure, diastolic blood pressure, smoking, diabetes mellitus, and eGFR [21]. High-, medium-, and low-risk were classified as Suita scores ≥ 56, 41–55, and <40, respectively. The estimated risks of developing coronary heart disease in 10 years in high, medium-, and low-risk groups were >9%, 2–9%, and <2%, respectively.

### 2.4. Outcome Data

Follow-up information was obtained from a review of medical records or telephone interviews blinded to the CAVI data. Major adverse cardiovascular events (MACE) included cardiovascular death, nonfatal myocardial infarction, nonfatal stroke, coronary revascularization, and heart failure requiring hospitalization. Strokes included ischemic and hemorrhagic strokes. The definitions of MACE have been previously described [6]. The time to the first primary endpoint was retrospectively evaluated.

### 2.5. Statistical Analysis

Data are expressed as mean ± standard deviation. Dichotomous variables are expressed as numbers and percentages. Categorical data were compared using the χ^2^ analysis or Fisher’s exact test. One-way analysis of variance was used to compare normally distributed continuous variables, and Bonferroni correction was used for post hoc testing. The relationship between continuous variables was investigated using Spearman’s correlation coefficient. Cumulative survival estimates were calculated using the Kaplan–Meier method and compared using the log-rank test. To ascertain the association between CAVI and MACE, we performed univariate and multivariate Cox regression analyses, and the results were reported as hazard ratios (HRs) with 95% confidence intervals (CI). Multivariate Cox regression analysis included variables with *p* < 0.05 in the univariate analysis. The incremental prognostic value of CAVI was assessed using receiver operating characteristic (ROC) curve analysis, continuous net reclassification improvement, and integrated discrimination improvement. All reported *p*-values were two-sided, and statistical significance was set at *p* < 0.05. Statistical analyses were performed using SPSS statistical software (version 28; IBM Corp., Armonk, NY, USA) and the R statistical package (version 3.5.2; R Foundation for Statistical Computing, Vienna, Austria).

## 3. Results

### 3.1. Comparison of Baseline Characteristics

The mean age of the patients was 68 ± 9 years, and 64% were male. The average CAVI was 8.8 ± 1.3. The baseline characteristics of 554 patients according to CAVI (normal group, CAVI < 8; borderline group, CAVI 8.0–9.0; abnormal group, CAVI > 9.0) are shown in Table 1. Patients with higher CAVI levels were older and more likely to be male. The mean systolic blood pressure and the prevalence of hypertension increased significantly with higher CAVI. The mean diastolic pressure, HDL-C levels, triglyceride levels, hemoglobinA1c, the prevalence of diabetes mellitus, dyslipidemia, smoking habits, and use of statins did not differ among the groups.

### 3.2. Association between Cumulative Incidence of Major Adverse Cardiovascular Events (MACE) and CAVI

**Figure 1:**
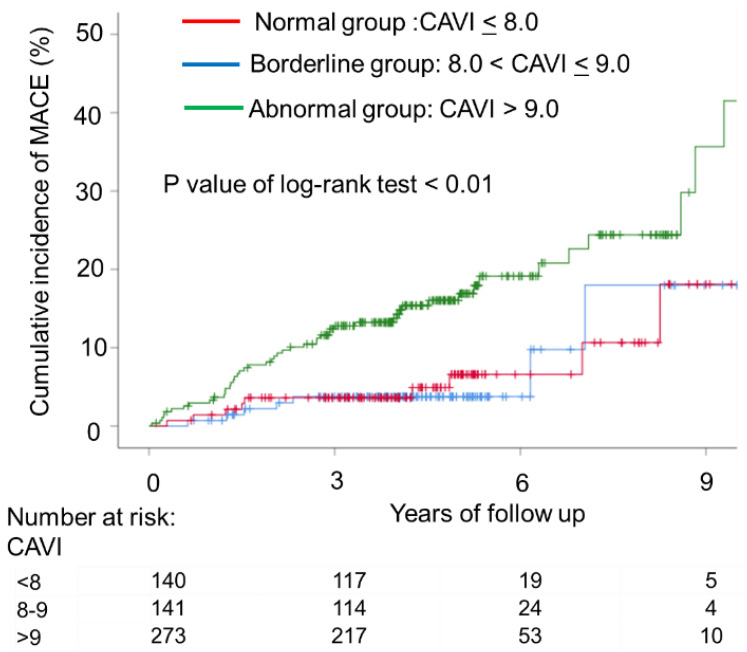
Kaplan–Meier plot of cumulative probability of cardiovascular events by cardio-ankle vascular index (CAVI) levels. Time to cardiovascular events, including cardiovascular death, nonfatal stroke, nonfatal myocardial infarction, heart failure requiring hospitalization, and coronary revascularization, according to baseline CAVI. The cumulative incidence rates of cardiovascular events according to CAVI levels were significantly higher in the abnormal group (CAVI > 9.0) than in the normal (CAVI < 8) and abnormal groups (8 < CAVI ≤ 9) (*p*-value for trend < 0.01).

**Figure 2:**
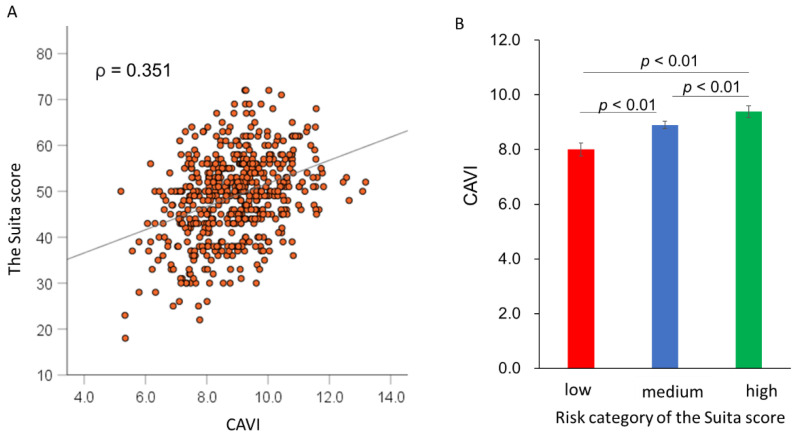
Correlation between CAVI and the Suita score. Scatter plot showing the correlation between CAVI and the Suita score (**A**) and CAVI according to the risk category of the Suita score (**B**).

During this follow-up period (median 4.3 years), 65 patients had MACE, including cardiac death (*n* = 2), myocardial infarction (*n* = 3), stroke (*n* = 13), heart failure with hospitalization (*n* = 14), or coronary revascularization (*n* = 34). The cumulative incidence rates of MACE according to CAVI levels are shown in Figure 1, and the rates were significantly higher in the abnormal group than in the other groups (*p*-value for trend < 0.001). Figure 2 shows the ROC curve analysis of CAVI for predicting MACE. The sensitivity and specificity of CAVI at a cutoff value of 9.0 were 75% and 54%, respectively (area under the curve, 0.688; *p* < 0.001). The multivariable-adjusted Cox proportional hazard model, CAVI (>9.0), was associated with an increased risk of MACE after adjusting for covariates (HR, 1.941 [95% CI, 1.092–3.448]; *p* = 0.024) (Table 2).

### 3.3. Incremental Predictive Value of CAVI over the Suita Score

**Figure 3:**
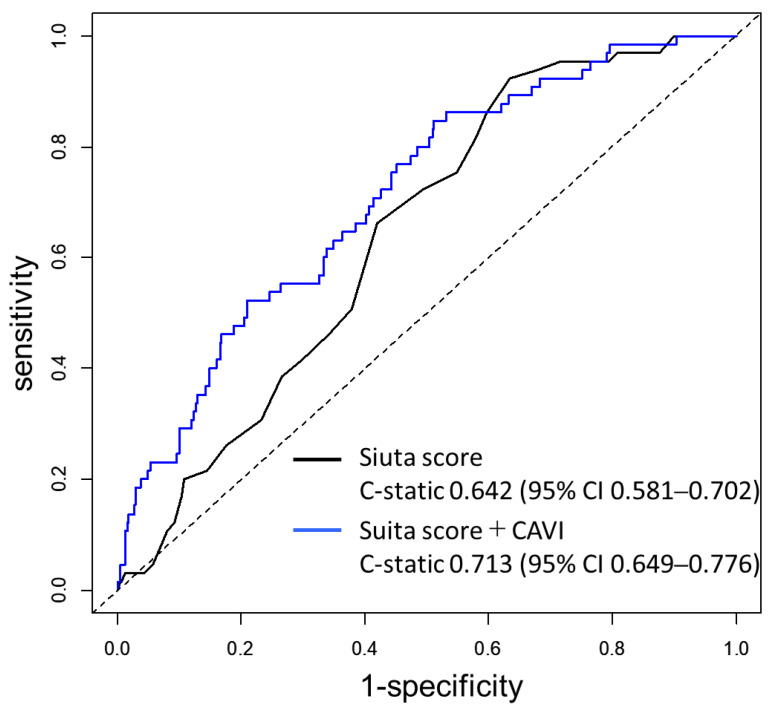
Comparison of the receiver-operating characteristic (ROC) curves. Black curve: predictive ability based on the Suita score. Blue curve: predictive ability based on the Suita score and CAVI. CI: confidence interval.

As shown in Figure 2A, the CAVI was weakly correlated with the Suita score (ρ = 0.351, *p* < 0.01). When patients were classified into three groups (low-risk, medium-risk, and high-risk groups) according to the Suita score, the CAVI value increased stepwise from the low-risk group to the high-risk group (8.1 ± 0.2, 8.9 ± 0.1, and 9.3 ± 0.2, respectively; *p*-value for trend < 0.01) (Figure 2B). An ROC curve analysis was performed to determine the incremental value of CAVI for predicting MACE (Figure 3). The addition of CAVI to the Suita score significantly improved the C statics from 0.642 to 0.713 (*p* = 0.04). The addition of the CAVI yielded a continuous net reclassification index of 0.293 (95% CI, 0.036–0.551; *p* = 0.025) and an integrated discrimination improvement of 0.0479 (95% CI, 0.0218–0.0740; *p* < 0.001).

## 4. Discussion

This study demonstrated that abnormal CAVI (>9.0) was significantly associated with the incidence of MACE in patients with CVD risk factors but without known CVD. Furthermore, the CAVI and Suita scores improved the prediction of MACE in these patients. To our knowledge, this is the largest study showing the incremental value of CAVI in addition to a clinical risk score for predicting cardiovascular events in the setting of primary prevention.

Several studies have shown that CAVI is associated with the incidence of cardiovascular events in patients with known CVD [5,6,7,8,9] and without CVD [10,11,12,13,14,15,16]. However, evidence on the incremental value of CAVI over a clinical risk score for predicting cardiovascular events has been limited. Satoh-Asahara et al. showed that in 300 obese patients without CVD, CAVI, in addition to the atherosclerotic cardiovascular disease risk score, moderately improved the prediction of cardiovascular events [16]. We showed an incremental value of CAVI for predicting cardiovascular events in a large cohort study, where one-third of the participants had a history of CVD [6]. The present study is in line with these two previous studies and clearly demonstrates the usefulness of CAVI for predicting cardiovascular events in patients with CVD risk factors but without known CVD.

This study showed that the best cutoff value of CAVI for predicting cardiovascular events was 9.0, which was consistent with our proposal for abnormal CAVI (>9.0) [17]. However, Satoh-Asahara et al. showed that, including 300 obese patients, the threshold of CAVI for cardiovascular events was 7.8 [16]. Compared to the study by Satoh-Asahara et al., the patients in our study were heterogeneous with hypertension, diabetes, or dyslipidemia who visited the Department of Cardiovascular Medicine. In addition, the mean age in their study was 52 years, which was lower than that in our study (mean age = 66 years). Therefore, these differences may be the reason for the higher cutoff values in the present study. Although our study validated that a CAVI of 9.0 was a cutoff for predicting cardiovascular events in the setting of primary prevention, further research is needed to confirm the optimal threshold of CAVI to predict cardiovascular events in each population.

Several measures of arterial stiffness, such as carotid-femoral PWV and brachial-ankle PWV, have been introduced [1]. However, notable differences were observed among the arterial stiffness measurements. Carotid-femoral PWV is obtained by applanation tonometry, which is a complicated technique compared with CAVI and brachial-ankle PWV [3]. CAVI and brachial-ankle PWV are automatically derived from a plethysmography cuff [1]. CAVI has an advantage over PWV for measuring arterial stiffness because it is less dependent on blood pressure at the time of measurement [4]. A reproducible assessment of arterial properties may allow detailed monitoring of changes in arterial stiffness in clinical practice. CAVI can be easily obtained automatically with a device, leading to its widespread use in clinical situations if cost constraints are ignored. Further investigations are needed to elucidate this matter, with due consideration given to cost-effectiveness.

This study has several limitations. First, we acknowledge that an observational study cannot definitively prove that there is a causal link underlying the association between increased CAVI and increased CVD events. Second, the study population included only Asian individuals. Although several studies on non-Asian populations have recently been reported [12,14], the generalizability of our data to other races/ethnicities remains uncertain. Third, we failed to estimate the cutoff value of CAVI for each event because of the small number of events. Hence, further studies with larger sample sizes are required.

## 5. Conclusions

We demonstrated that abnormal CAVI (>9) was significantly associated with the incidence of cardiovascular events in patients with CVD risk factors but without known CVD. Furthermore, CAVI in addition to the Suita score improved the prediction of cardiovascular events in these patients. The data in this study suggest that the measurement of CAVI is a clinically useful means to predict the development of cardiovascular events in the setting of primary prevention.

## Figures and Tables

**Table 1:** Baseline Characteristics According to the CAVI.

	All	Normal CAVI	Borderline CAVI	Abnormal CAVI	
		(CAVI < 8)	(8 ≤ CAVI < 9)	(9 ≤ CAVI)	
Variables	(*n* = 554)	(*n* = 140)	(*n* = 141)	(*n* = 273)	*p*-Value for Trend
Age, year	66 ± 9	62 ± 10	67 ± 9	71 ± 7	<0.01
Male, *n* (%)	353 (64)	92 (66)	76 (54)	185 (68)	0.01
Body mass index, kg/m^2^	23.4 ± 3.9	24.7 ± 4.6	23.9 ± 3.8	22.9 ± 3.5	<0.01
Hypertension, *n* (%)	418 (75)	84 (60)	110 (78)	224 (82)	<0.01
Diabetes mellitus, *n* (%)	283 (51)	69 (43)	76 (54)	138 (51)	0.72
Dyslipidemia, *n* (%)	343 (62)	82 (59)	92 (65)	169 (62)	0.52
Current smoking, *n* (%)	159 (29)	44 (31)	41 (29)	74 (27)	0.65
Systolic blood pressure, mmHg	129 ± 19	122 ± 19	128 ± 16	133 ± 18	<0.01
Diastolic blood pressure, mmHg	74 ± 11	72 ± 11	74 ± 10	75 ± 11	0.04
Laboratory data					
Triglyceride, mg/dL	140 ± 119	136 ± 126	137 ± 94	144 ± 126	0.78
HDL-C, mg/dL	55 ± 17	58 ± 19	54 ± 18	545 ± 16	0.08
LDL-C, mg/dL	109 ± 31	113 ± 33	110 ± 31	107 ± 32	0.17
HemoglobinA1c, %	6.4 ± 1.4	6.3 ± 1.5	6.5 ± 1.4	6.5 ± 1.4	0.69
eGFR, mL/min/1.73 m^2^	66.1 ± 19.4	70.0 ± 17.3	68.0 ± 20.9	63.3 ± 18.2	0.02
Medications					
Antihypertensive agents, *n* (%)	406 (73)	85 (61)	107 (76)	214 (78)	<0.01
Antidiabetic agents, *n* (%)	178 (42)	34 (37)	52(47)	92 (42)	0.31
Lipid-lowering agents, *n* (%)	310 (56)	64 (46)	86 (61)	160 (59)	0.01

HDL-C, high-density lipoprotein cholesterol; LDL-C, low-density lipoprotein cholesterol; eGFR, estimated glomerular filtration rate.

**Table 2:** Association Between the CAVI and Cardiovascular Events.

	Univariate		Multivariate	
	HR (95% CI)	*p*-Value	HR (95% CI)	*p*-Value
CAVI > 9	3.07 (1.78–5.41)	<0.01	2.31 (1.27–4.18)	<0.01
Age per year	1.05 (1.01–1.08)	<0.01	1.02 (0.98–1.06)	0.18
Male	1.97 (1.09–3.57)	0.02	1.86 (1.02–3.39)	0.04
Hypertension	2.56 (1.16–5.62)	0.01	0.86 (0.38–2.27)	0.86
Diabetes mellitus	1.44 (0.87–2.37)	0.14		
Dyslipidemia	1.68 (0.96–2.93)	0.06		
Current smoking	1.34 (0.80–2.25)	0.25		
Antihypertensive agents	5.36 (1.94–14.78)	<0.01	4.16 (1.29–13.40)	0.01
Antidiabetic agents	1.41 (0.32–2.40)	0.20		
Lipid-lowering agents	1.79 (1.05–3.07)	0.03	1.56 (0.91–2.68)	0.10

The multivariate analysis included CAVI > 9, age, male sex, hypertension, antihypertensive agents, and lipid-lowering agents. CAVI, cardio-ankle vascular index; HR, hazard ratio; CI, confidence interval.

## Data Availability

The data presented in this study are available upon request from the corresponding authors. The data were not publicly available because of privacy concerns.
